# Sex-biased patterns shaped the genetic history of Roma

**DOI:** 10.1038/s41598-020-71066-y

**Published:** 2020-09-02

**Authors:** C. García-Fernández, N. Font-Porterias, V. Kučinskas, E. Sukarova-Stefanovska, H. Pamjav, H. Makukh, B. Dobon, J. Bertranpetit, M. G. Netea, F. Calafell, D. Comas

**Affiliations:** 1 Institute of Evolutionary Biology (UPF-CSIC), Department of Experimental and Health Sciences, Universitat Pompeu Fabra, Barcelona, Spain; 2 Department of Human and Medical Genetics, Faculty of Medicine, Biomedical Science Institute, Vilnius University, Vilnius, Lithuania; 3 Research Center for Genetic Engineering and Biotechnology “Georgi D. Efremov”, Academy of Sciences and Arts of the Republic of North Macedonia – MASA, Skopje, Republic of North Macedonia; 4 Institute of Forensic Genetics, Hungarian Institute for Forensic Sciences, Budapest, Hungary; 5 Institute of Hereditary Pathology, Ukrainian Academy of Medical Sciences, Lviv, Ukraine; 6 Department of Internal Medicine and Radboud Center for Infectious Diseases, Radboud University Medical Center, 6525 GA Nijmegen, the Netherlands; 7 Department of Human Genetics, University of Medicine and Pharmacy Craiova, Craiova, Romania; 8 Department for Genomics and Immunoregulation, Life and Medical Sciences Institute (LIMES), University of Bonn, 53115 Bonn, Germany

**Keywords:** Genetic variation, Genetic variation

## Abstract

The Roma population is a European ethnic minority characterized by recent and multiple dispersals and founder effects. After their origin in South Asia around 1,500 years ago, they migrated West. In Europe, they diverged into ethnolinguistically distinct migrant groups that spread across the continent. Previous genetic studies based on genome-wide data and uniparental markers detected Roma founder events and West-Eurasian gene flow. However, to the best of our knowledge, it has not been assessed whether these demographic processes have equally affected both sexes in the population. The present study uses the largest and most comprehensive dataset of complete mitochondrial and Y chromosome Roma sequences to unravel the sex-biased patterns that have shaped their genetic history. The results show that the Roma maternal genetic pool carries a higher lineage diversity from South Asia, as opposed to a single paternal South Asian lineage. Nonetheless, the European gene flow events mainly occurred through the maternal lineages; however, a signal of this gene flow is also traceable in the paternal lineages. We also detect a higher female migration rate among European Roma groups. Altogether, these results suggest that sociocultural factors influenced the emergence of sex-biased genetic patterns at global and local scales in the Roma population through time.

## Introduction

The Roma are the largest and most widespread ethnic minority in Europe. However, given the paucity of written records, it is also one of the least documented within the continent^1,2^. Genetic, linguistic and cultural evidence points to the Roma having originated in North-Western India ~ 1,500 years ago (ya) from a low number of proto-Roma founders^3–5^. These founders started a diaspora through West Asia, and arrived for the first time in the European continent at the Balkan Peninsula ~ 1,000 ya^1,2^. Most European Roma became sedentary in the Balkans, while nomadic groups spread through Europe: Vlax Roma moved into the Danubian Principalities (currently Romania, Moldova and parts of Hungary); Romungro Roma spread within the Austro-Hungarian Empire; and North-Western Roma continued moving to North and West Europe. In addition to the nomadic nature of this population, a history of continuous persecution and social exclusion triggered wide dispersals within Europe. These dispersals led gradually to the formation of different ethnolinguistic groups (*i.e.*migrant groups), turning the Roma into a mosaic of diverse subpopulations^1,2^.

The migration out of India of the proto-Roma left strong traces of a founder effect in their genomes. This has been observed as a drastic decrease on their effective population size (Ne), half that of the source population^3,4^. During their diaspora and settlement, the extensive gene flow with non-Roma groups contributed to the formation of present-day Roma autosomal genomes, which are a mixture of South Asian and West Eurasian components^3,4^, at 35% and 65% frequencies, respectively^6^. Uniparental marker studies have added deeper layers of resolution identifying specific lineages of these components in the Roma population: comparing them with non-Roma male and female populations, they show that Roma experienced extensive drift and have a lower Ne as a result of a series of bottlenecks during their diaspora^7,8^. The specific lineages found revealed a higher gene flow from non-Roma to Roma groups, and confirmed their origin in the Northwest of the Indian subcontinent. The possibility to trace a geographic origin to a specific uniparental lineage is a powerful tool to infer recent demographic events that are not always straightforwardly detected with genome-wide analyses^9^. However, as these lineages show low frequencies or are absent in non-Roma populations, they have not been properly described.

The comparison of mtDNA and the male-specific portion of the Y chromosome (MSY) is of special interest in order to reveal sex-biased genetic patterns in human populations^10^, since parameters contributing to population evolution such as generation time, migration rates and admixture can contribute differently to the two non-recombining markers^9^. These asymmetric processes can be traced at local and regional levels, although their footprint is less evident at a global scale^11,12^, as seen in multiple genetic studies from a wide range of human populations: Madagascar shows a different geographic distribution for the maternal and paternal source of Indonesian ancestry^13^; gene flow in South and Central American populations appears to be mediated by paternal European lineages and maternal Native American and African ones^14–16^; in Thailand, female dispersal rate is higher than male in patrilocal groups, whereas, in matrilocal populations, an equal exchange is observed^17^. Finally, in South Asia, mitogenomes have been conserved since the first pre-Holocene settlements, but a replacement of Y chromosomes occurred with subsequent Bronze Age migrations^18^. In the Roma, sociocultural traditions and a previous study in local Bulgarian Vlax groups^19^ suggest a sex-biased history. However, the scope, timespan and potential impact on their genetic landscape have not been characterized yet at a larger scale.

In this study, we compared 76 complete mtDNA and MSY European Roma sequences in order to assess whether the Roma have undergone sex-biased processes at different population and genetic levels. To achieve this, we assayed asymmetric patterns regarding Roma as a whole, testing its origin and influence from external sources. We also performed a higher resolution analysis within Roma, looking into putative unbalanced genetic diversity and substructure.

## Results

### Roma genetic diversity is the result of a sex-biased complex demographic history

In order to unravel the demographic history of the Roma uniparental markers, we analysed 76 European Roma samples (Supplementary Table S1) together with 126 non-Roma European, West Asian, North African, and South Asian samples (Supplementary Table S2, Supplementary Figure S1).

Although the Roma are an admixed population between West Eurasian and South Asian groups, their mtDNA and MSY sequences show significantly high φst values with European, West Asian, and South Asian populations, together with lower diversity levels (Supplementary Figure S2, Supplementary Table S3). This suggests that the proto-Roma bottleneck and subsequent genetic drift had an impact on their uniparental genomes, differentiating them from the source populations.

**Figure 1:**
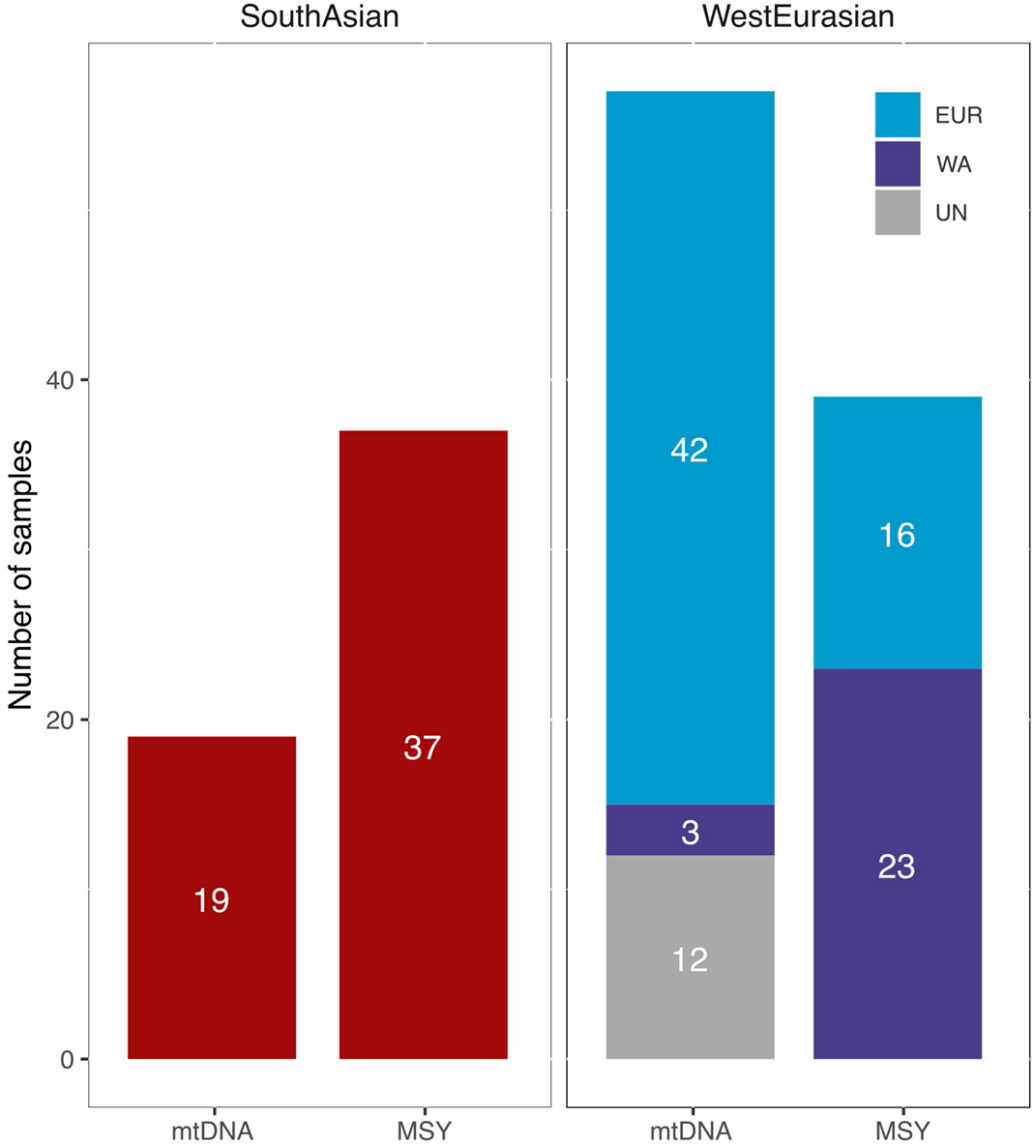
Number of samples of each origin for mtDNA and MSY lineages. West Eurasian lineages are further subdivided into European (EUR), West Asian (WA), and unassigned (UN).

However, these demographic events have led to different maternal and paternal genetic profiles in present-day Roma. Their composition in terms of lineage origins is significantly different between mtDNA and MSY (*p* value = 10^−9^) (Fig. 1, Supplementary Table S4). In particular, South Asian lineages are more frequent in the MSY than in the mtDNA sequences (49% and 25%, respectively), whereas mtDNA has a higher proportion of West Eurasian lineages compared to MSY. Moreover, the West Eurasian fraction presents different distributions, being European lineages predominant in mtDNA (73, or 95% if we assume that the unknown lineages are European) and West Asian in MSY (59%) (Fig. 1). The mtDNA and MSY lineage origin within the same individual are not associated (*p* value = 0.186) (Supplementary Table S5). Taken together, these results seem to indicate that sex-biased genetic patterns could have been already present in the proto-Roma population or started to appear right after their diaspora.

**Figure 2:**
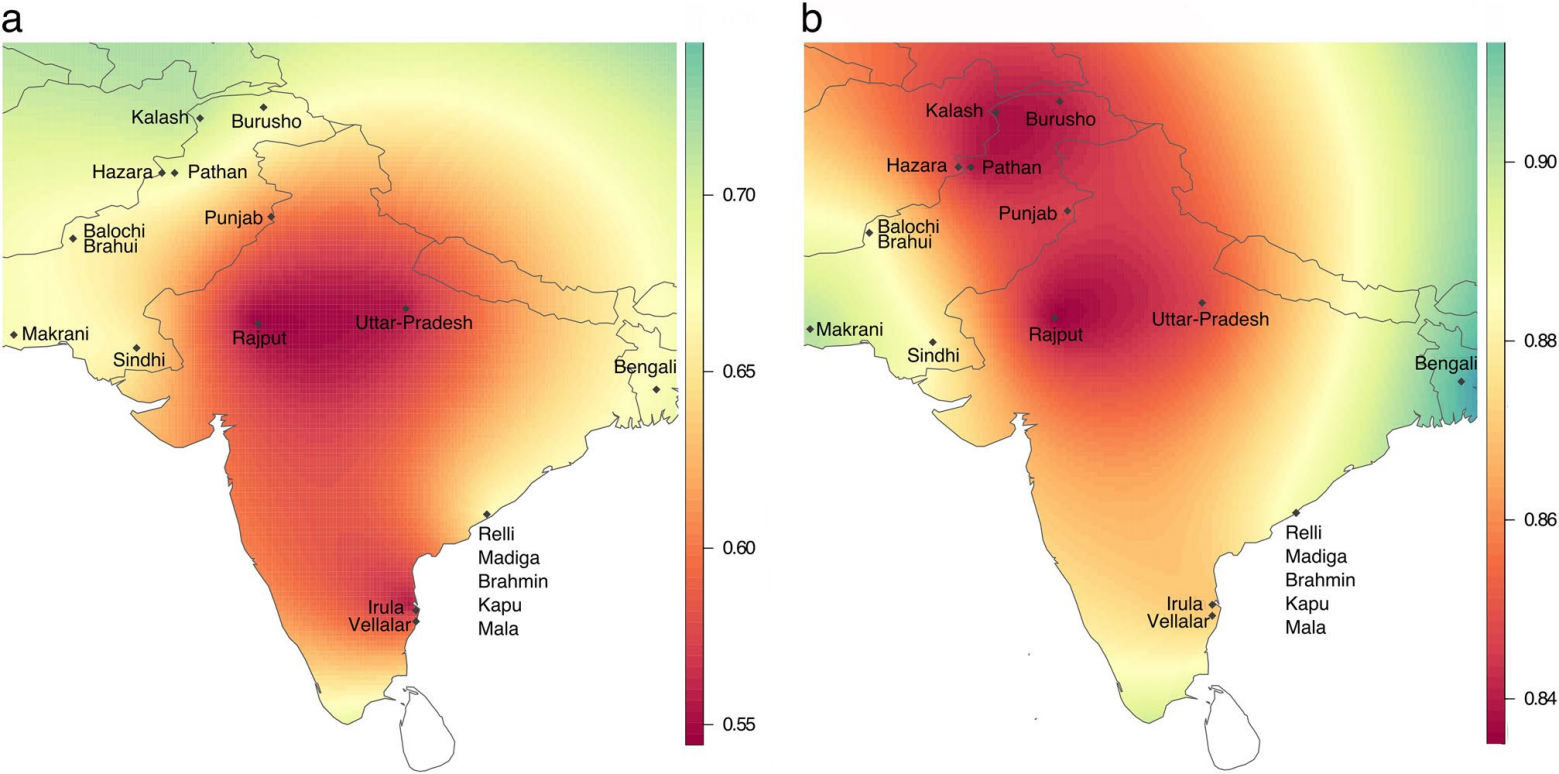
Spatial distributions of φst distances between Roma (only samples with a South Asian lineage origin) and South Asian populations in mtDNA (**a**) and MSY (**b**).

**Figure 3:**
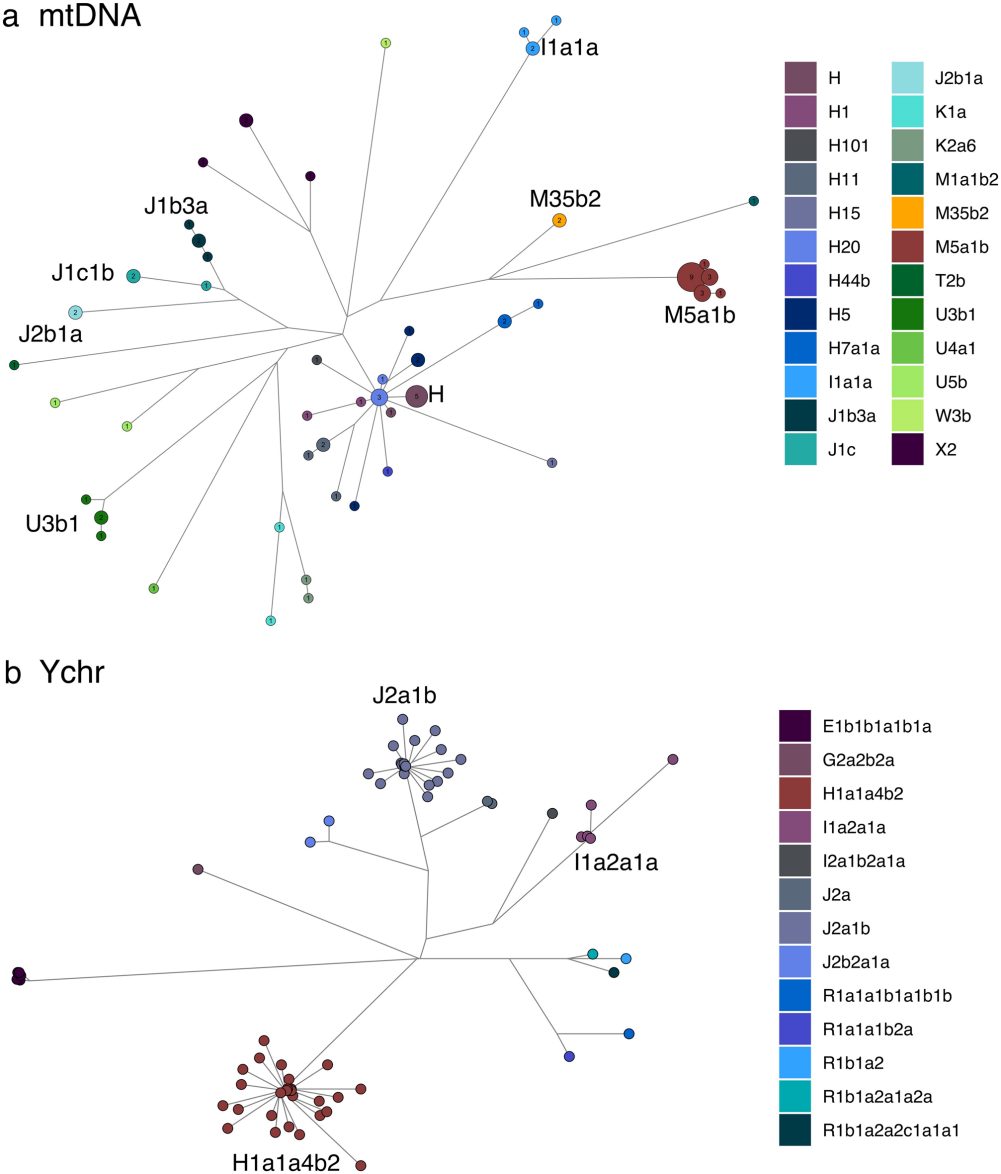
Reduced median joining networks for mtDNA coding region (**a**) and MSY (**b**) Roma samples, colored by haplogroup with main lineage labels.

In order to assess the origin and composition of the proto-Roma lineages, we analyzed the mtDNA and MSY lineages with a South Asian origin found in our Roma samples (Supplementary Table S2). Both uniparental markers present different genetic distances with the reference South Asian populations: mtDNA has a more widespread pattern exclusive to India, whereas MSY shows lower φst values limited to Northwest India and Pakistan (Fig. 2). A contribution of South Indian groups to the mitogenome pool is suggested also in our results (Fig. 2A). However, this outcome could be influenced by the lower phylogeographic resolution of the mtDNA compared to the available MSY, due to its smaller sequence size (15,569 bp vs 8.97 Mbp). Furthermore, two mtDNA South Asian lineages, M35b2 and M5a1b^20^, might have diverged before the migration out of India (Fig. 3A, Supplementary Table S6). In contrast, the single MSY lineage present in the Roma that went out of India, H1a1a4b2^21^, has recent divergent sub-branches private to the Roma population (Fig. 3B, Supplementary Table S6). Besides these differences, the migration out of India has left genetic traces in both uniparental makers, as evidenced by the star-like divergence patterns in the M5a1b and H1a1a4b2 Roma lineages (Fig. 3). The analysis of Ne dynamics reveals a flat Bayesian skyline plot (BSP) for the South Asian Roma mtDNA lineages (Supplementary Fig. S3A-B), although the low sample size and number of segregating sites might mask the changes in population size, and the Ne absolute numbers are not comparable^22^. MSY BSP shows a continuous expansion after the diaspora out of India (around 1,500 ya) (Supplementary Figure S3C).

During their diaspora and subsequent settlement, Roma experienced an extensive West Eurasian gene flow, reflected by more than half of uniparental lineages having a non-South Asian origin (Fig. 1). This pattern is even more pronounced in the mitogenomes, where there are several divergent European lineages with ancient coalescence ages. In contrast to the South Asian lineages, mtDNA haplogroups acquired through admixture do not present star-like expansion patterns inside Roma (Fig. 3A, Supplementary Table S6). Most of the non-Indian MSY sequences belong to two main lineages putatively introduced in two different admixture events: J2a1b from West Asia^7,23^, and I1a2a1a from Europe^7^ (Fig. 3B). Both haplogroups show star-like networks (Fig. 3B) with recent divergence within Roma males (Supplementary Table S6).

Taken together, these results show that the uniparental genetic landscape that we observe nowadays in Roma people is a consequence of a sex-biased foundation of the proto-Roma and asymmetric gene flow events. Incorporations of non-Roma females are detected as independent events spread through time while male admixture pulses are less frequent but with a higher influx rate.

### Uniparental genomes reveal different subpopulation structure within Roma

We then studied whether genetic diversity within Roma groups follows the same structure in both markers. Previous studies suggested that the Roma population is genetically structured by migration route^7,24^. Our AMOVA results for mtDNA showed significant values in each classification we tested (see “Methods”), with country of residence explaining the highest percentage of variance (4.79%, *p* value = 0.019) (Supplementary Table S7). This suggests that migrant groups do not act as maternal genetic boundaries. On the contrary, the grouping criterion that explained significantly more Y sequence diversity was the combination of migrant route and country of residence (7.88%, *p* value = 0.035) (Supplementary Table SS7). These genetic groups cannot be explained by differences in their molecular diversity indexes, as all Roma groups have overlapping confidence intervals for both uniparental markers (Supplementary Table S8).

To further explore the genetic substructure within Roma groups, Multidimensional Scaling (MDS) analyses were performed from φst distances (Supplementary Figure S4). Distinct substructure patterns were found between both markers, as evidenced by the lack in correlation between their genetic distances (R^2^ = −0.193, *p* value = 0.7219) ( Supplementary Figure S5). In fact, different migrant groups within the same country of residence (*i.e.* Hungarian Vlax and Romungro) clustered closer in the mtDNA MDS than in the MSY analysis (Supplementary Figure S4), which may explain the AMOVA results.

**Figure 4:**
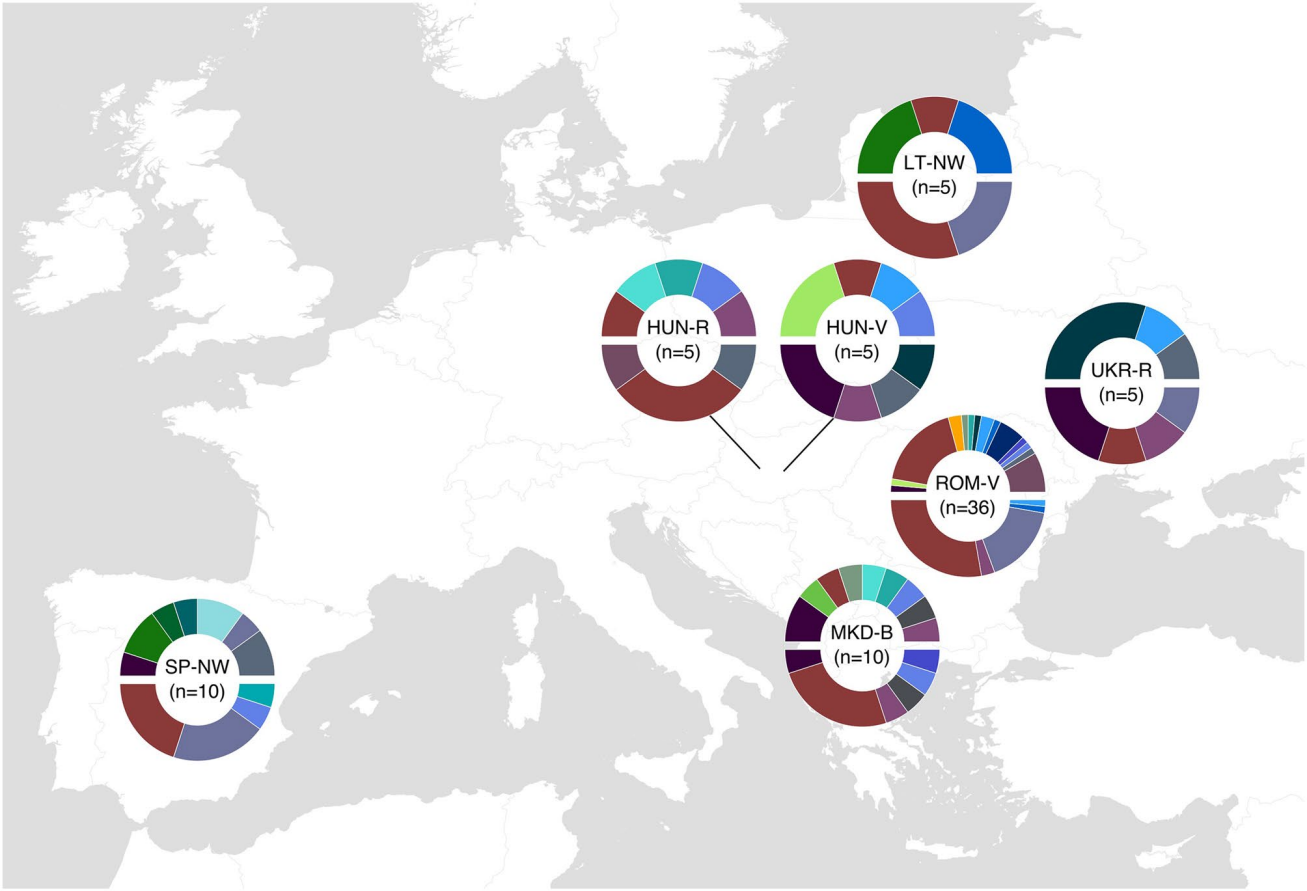
Haplogroup distribution for each Roma group colored by origin (i.e. red colors for South Asian, blues and greens for West Eurasian). mtDNA lineages in the upper charts and MSY in the lower. Haplogroups are colored as in Fig. 3. SP-NW: Spain North-West; MKD-B: Macedonia Balkan; ROM-V: Romania Vlax; UKR-R: Ukraine Romungro; HUN-V: Hungary Vlax; HUN-R: Hungary Romungro; LT-NW: Lithuania North-West.

Focusing on the haplogroup composition as a driving force for Roma substructure, we observe common group-specific patterns: the Macedonia-Balkan group shows the highest haplogroup diversity for both markers, and North-Western groups (Lithuania and Spain) share a specific mtDNA haplogroup (U3b1) and similar MSY haplogroup frequencies (Fig. 4). Additionally, the sex bias observed between mtDNA and MSY lineage proportions in the Roma as a population is also found when focusing in subgroups: numerous mtDNA European haplogroups are present in each group, while the single South Asian MSY haplogroup is widely spread in all of them.

Considering our results, a different population substructure is detected when comparing mtDNA and MSY within Roma, where present-day migration group affiliation might act as a barrier only for male migration. However, Roma groups from different countries that shared the migration route experienced the same genetic drift that increased the frequency of some haplogroups, as shown by the common lineage composition in the North-Western group.

### Roma lineages show hidden phylogenetic complexity

#### mtDNA haplogroups

Mitochondrial haplogroup M5a1b is present in 17 Roma samples, which also have the 3954 T-9833C coding motif, enabling us to subclassify them as M5a1b1a^25^. Moreover, 13 out of the 17 individuals share the control region polymorphism 16298C, defining M5a1b1a1^25^, together with previously published 105 M5a1 Roma^7^ and 6 Punjabi samples^26,27^. The remaining 4 Roma samples (3 unique sequences) can be grouped into a new lineage (M5a1b1a2), defined by the absence of 16298C, but with the coding 15902C variant, also present in 4 South Asian samples^26,27^ (Supplementary Figure S6A). The fact that Roma and South Asians share variants within this haplogroup might indicate that two different M5a1b lineages (*i.e.* M5a1b1a1, M5a1b1a2) were already present in Indian groups before the proto-Roma left India.

Within the European lineages, 5 Romanian Vlax mitogenomes (3 unique sequences) are classified with the basal haplogroup H, although they share the 1271G-3621C-16223T motif, defining a new H lineage which would be named as H107.

#### MSY haplogroups

Formerly undescribed phylogenetic variants are detected in the three main Roma lineages. Within H1a-M2853, we describe a new Roma-specific branch defined by 18 polymorphisms, absent in the reference panel populations, which we propose to call H1a1a4b2d (Supplementary Table S9). Moreover, three sub-branches of H1a1a4b2d are observed: the first formed by 6 Vlax samples and defined by 6 SNPs, the second group with 7 individuals and 2 common mutations, and the last sub-branch defined by 26618569C carried by 6 males. Within the last sub-branch, 3 private variants are present in a group of 3 Spanish Roma (Supplementary Figure S6B).

Inside the J2a-M92 clade, we detected 5 private SNPs markers common to all 18 samples (Supplementary Table S9). Lastly, for the entire lineage I1a-Z62, a total of 17 previously unknown and exclusive polymorphic positions were discovered (Supplementary Table S9).

This phylogenetic refinement revealed an unprecedented complexity in the proto-Roma uniparental gene pool.

## Discussion

The South Asian origin and West Eurasian admixture of Roma are well known^3,4^. However, the high values of population differentiation (φst) that we found between Roma and the reference populations (Supplementary Fig. S2) are evidence of a genetic landscape shaped by a further complex demographic history. We found a series of sex-biased patterns and genetic variation in Roma. A trend towards the ancestral components being found at different proportions in male or female lineages has been detected in previous uniparental studies^7,24^, but here the availability of complete sequences for mtDNA and MSY revealed that South Asian lineages have been strongly maintained in the paternal fraction of the population (49%), whereas in mtDNA they appear to be diluted by higher gene flow from West Eurasian sources (75%). Moreover, the genetic contribution of the different regions within West Eurasia is not equal for both markers (Fig. 1). Our results show that the demographic history of Roma has been continuously sex-biased, strongly impacting their genomes since their South Asian origin, during their diaspora through West Eurasia and until their settlement in Europe.

The composition and foundation of the Roma source population that went out of India has been one of the main unresolved questions. Although a notable founder effect is observed in both their maternal and paternal South Asian lineages, the origins of the uniparental proto-Roma genomes appear to be slightly different. The populations currently in Pakistan contributed to the male component in Roma, but, interestingly there is no trace of female input from this region (Fig. 2). In fact, the Northwest origin of the H1-M2853 MSY lineage is confirmed, as it was previously reported for its paternal ancestor H1-M82^21^. The maternal diversity is instead higher, and it is a representation of the existing Indian genetic landscape, whereas the paternal diversity appeared synchronically with the Roma expansion. Our results show a higher variance and more diffuse origin of mitogenomes reflecting the presence of patrilocal patterns in the proto-Roma source population, with more homogeneous male genetic pools^28^.

The subsequent history of Roma outside South Asia followed sex-biased patterns as well. The West Eurasian proportions that we describe (Fig. 1) imply gene flow rates around 2.73% and 1.38% per generation, for the mtDNA and MSY respectively (assuming a raw constant gene flow during 50 generations). In addition, modest gene flow from Roma to non-Roma groups has been previously detected^7^, where footprints of specific Roma haplogroups have been identified in other European populations (*e.g.* the mtDNA M5 haplogroup in the general Spanish population^29^). This bidirectional gene flow, although at different rates, is higher than expected given the isolation and social exclusion of the Roma population^1,2^. Regarding the West Eurasian ancestry in the Roma, it can be further divided with a West Asian and a European fraction, both differentiated in terms of quantity and lineage ages. More than half of mtDNA sequences are of European origin (55% ± 16%); however, they belong to different haplogroups with distant coalescent ages (Supplementary Table S6) consistent with the history of each lineage in the external European populations. Independent and time spread incorporations of non-Roma females are the most likely source of the present-day diversity. In contrast, the highest MSY gene flow is from a West Asian source (30%), with 95% of the sequences belonging to a single haplogroup, J2a-M67. Although its parent lineage J2a-M410 has a frequency distribution that includes the Indian subcontinent, the J2a-M67 sub-branch is absent in this region and correlated instead with the spread of early farmers and Bronze Age cultures in Anatolia and the Mediterranean basin^23^. The star-like pattern in this haplogroup reveals a pulse of admixture during the Roma journey through West Asia. The European male influence is mostly represented by the I1a-Z140 lineage, of Balkan origin^7^, and few sporadic and differentiated events. These results might suggest that the inclusion of European females into Roma has been traditionally easier, while male incorporations might have been restricted to certain specific episodes that might be related to periods with changes in exclusion politics^1,2^. This genetic landscape is in accordance with the previously known social structure of Roma, where sociocultural group affiliation is patrilinearly transmitted^30^, a common feature in most human societies^31,32^. Although this pattern does not appear in the West Asian ancestry, the putative signal could have been diluted in mtDNA because of the larger European gene-flow and lower resolution.

We further explored to which extent the mentioned sex bias had influenced Roma internal genetic structure. A non-geography-based grouping has been proposed for the Roma population, where genetic variation is more closely associated to sociocultural groups (migrant groups)^7,24^. Here, we show that this hypothesis does not apply for uniparental ancestry: actually, in both non-recombining markers, migrant group affiliation alone does not explain the present diversity when considering all Roma groups (Supplementary Table S7). Despite this, the similarity found between the two most distant subpopulations (Lithuanian and Spanish) hints at a shared migrant history and differentiation specific for North-Western Roma, as previously suggested^8,24^. MDS analyses and lower φst distances between mtDNA Roma groups point to an easier migration of females among Roma subpopulations (Supplementary Figure S4), mirroring the patrilocality effects found in other human populations with similar social constructions^33^. Moreover, the lack of correlation between mtDNA and MSY φst within groups suggests that the sex-biased genetic landscape that we have found in Roma as a whole population is present also when observing their internal structure. Additional samples would be required for a more precise characterization of the individual demographic evolution of Roma subpopulations.

The addition of our complete sequences for mtDNA and MSY allowed us to refine the Roma phylogeny. The new South Asian mtDNA sublineage M5a1b1a2 (Supplementary Figure S6) is a sister branch of the previously described M5a1b1a1^25^. The presence of its motif mutations in South Asian individuals points to a divergence time previous to the out of India migration. Diversity in the MSY lineages originated instead within the Roma and it is extremely recent, likely related to the population growth after their arrival to Europe (Supplementary Table S6). In detail, H1a1a4b2 presents a complex internal structure with at least three sub-branches private to Roma males (Supplementary Figure S6). Similarly, as it shown in the mtDNA, in the MSY one of the new clades is specific for Romanian Vlax: as this is the group with more individuals in our dataset (n = 36), we suggest that additional subpopulation related lineages could appear in other Roma groups when increasing sample size. Additionally, another of these sister clades is mostly formed by North-Western individuals and has an internal subclade of Spanish Roma. These results confirm North-Western Roma as the migrant group with the highest shared genetic background.

In the present study, we have unraveled the sex-biased patterns that have shaped the genetic history of Roma, using the highest possible resolution in mtDNA and MSY sequences. Multiple genetic features point to an asymmetric genetic variation when comparing both uniparental markers. Within these, the most important are the lineage diversity within proto-Roma, the gene flow proportions from West Eurasian non-Roma populations, and the internal genetic structure within Roma groups. These results suggest that the Roma sociocultural system, together with the European exclusion politics, influenced the emergence of these genetic patterns both at global and local scales. However, social structure dynamics is not an immutable entity and past patrilocal and patrilineal systems might be reflected in the present-day genetic landscape of the Roma people.

## Methods

### Samples

We collected 40 saliva samples from males in six European Roma populations (Lithuania, Spain, Macedonia Balkan, Ukraine Romungro, Hungary Romungro and Hungary Vlax). DNA was extracted using a standard phenol–chloroform procedure. Library preparation and sequencing were done in Macrogen Facility (Seoul, Korea), with TruSeq Nano DNA (350) kit and whole-genome shotgun paired-end sequencing (Illumina HiSeq X Ten) to a mean coverage of 29X (Supplementary Table S1). Roma individuals were selected for having all four grandparents belonging to the same geographical and sociocultural population. Additionally, 36 Roma males from Romania were included (Dobon B et al., in submission). As a reference panel, 28 European, 23 West Asian, 73 South Asian and 2 North African samples were used, only males were included in MSY analyses (Supplementary Table S2).

### Data processing

The complete mtDNA genomes were reconstructed using the mtArchitect pipeline^34^. This approach ensures mapping flexibility in highly variable regions and captures the maximum range of reads, avoiding the incorporation of nuclear mitochondrial regions (NUMTs). Two successive mapping steps (lax and iterative) are performed to create a modified mitochondrial reference for each sample, using Burrows-Wheeler Alignment (BWA) 0.7.15^35^, SAMtools 1.3.1^36^, Vcftools 0.1.12^37^ and the revised Cambridge Reference Sequence (rCRS)^38^. The original reads are mapped to the sample-specific modified reference and the GRCh38 nuclear reference, retaining only high-quality paired-end reads (mapping quality ≥ 50 and Phred quality score ≥ 33). Contigs are subsequently constructed from the reads whose depth of coverage is ≥ 150X using Hapsembler 1.1^39^. Lastly, the contigs from the de novo assemblies are oriented and joined with MAFFT 7.130b^40^, and the consensus sequence is used to reconstruct each mitogenome. Mitochondrial haplogroups were identified using Haplogrep^41^, based on phylotree build 17 (www.phylotree.org, 18 Feb 2016)^42^ (Supplementary Table S2).

To obtain the MSY sequences, all raw reads were mapped to the human reference sequence GRCh38 with BWA^35^. After selecting the mapped reads, we removed PCR duplicates, recalibrated base quality scores and performed the haplotype calling using the Genome Analysis Toolkit (GATK) v3.7–0^43^. Variants were called specifically for the MSY using the GATK GenotypeGVCFs^43^ tool. The polymorphisms underwent hard filtering according to GATK best practices recommendations^44^. Being the MSY especially rich in repeats, duplications and low-quality regions, we restricted our analysis to high quality regions as defined by Wei et al^45^. Together these comprise 8.97 Mb of unique Y chromosomal sequence, which were defined as the “callable region". As by the Mondal et al.^46^ procedure, only those positions with a total coverage (summed across individuals) between half and double of the average (654–2,616) were selected, resulting in two datasets, a first one with only Roma samples and 4,674 variants, and a second one with Roma and the reference panel of 12,973 variants. Haplogroups were called with yHaplo^47^ after a genomic coordinate liftover to GRCh37 (Supplementary Table S2).

The depth of coverage of each mtDNA genome and MSY was retrieved using GATK v3.7–0^43^ (Supplementary Table S1).

### Statistical analyses

Maximum likelihood (ML) phylogenetic trees for Roma and non-Roma mtDNA and MSY sequences were inferred with RAxML 8.2.4^48^ using the GTRCAT substitution model^49^ with 1,000 bootstrap runs; results were visualized in the ggtree R package^50^. Taking advantage of the haploidy of the markers, the origin of all Roma sequences (*i.e.* West Eurasian and South Asian) was defined using their cluster affiliations in the ML phylogenetic tree (Supplementary Figure S1) together with the haplogroup frequencies in the literature (Supplementary Table S2). In addition, West Eurasian lineages were further classified into European and West Asian, except in those haplogroups without a clear defined origin in the literature (Supplementary Table S2)^51–^^65^. χ^2^ tests were computed with the *stats* R package^66^ with the proportion of mtDNA and MSY lineage origins.

Analyses of molecular variance (AMOVA) were performed to test the correlation between the country of residence and the migration routes. We have tested three grouping scenarios independently: migration route (Balkan, Vlax, Romungro and North-Western), country of residence (Macedonia, Romania, Hungary, Ukraine, Lithuania and Spain), and both subdivisions combined (see Table S7). Random subsampling of 15 Romanian Vlax sequences was performed to compare Roma groups with balanced sample sizes. Molecular diversity indexes and φst (a measure for population differentiation based on nucleotide diversity) were calculated for mtDNA and MSY using Arlequin 3.5.2.2^67^. Geographic distributions of φst distances between South Asian Roma lineages and Pakistan and India reference populations were computed using the kriging model implemented in the *fields* R package^68^. Classical MDS analyses were performed with the *stats* R package^66^ for mtDNA and MSY φst matrices, independently. To test the correlation between mtDNA and MSY φst distances, Mantel tests were computed with the *ade4* R package with 10,000 permutations^69,70^.

Reduced median phylogenetic networks for Roma mtDNA and MSY sequences were constructed with Network 5.0.1.1 and visualized with the Network Publisher extension^71^. Specific mitochondrial sites were subtracted from the network analysis: known mutation hotspots and an insertion in positions 941–942, to avoid variable mutation rates^42,72^. The coalescence ages of haplogroups were estimated considering only the mitochondrial coding region (577–16,023) using a mutation rate of one nucleotide substitution every 3,533 years^73^. For the MSY estimations we used a fast mutation rate of 10^−9^ substitutions/year/site^74^, transformed in 1 mutation every 115 years in the callable portion of our dataset sequences (8.97 Mbp).

Bayesian evolutionary analyses were performed with BEAST2 2.5.0^75^. BSP were constructed for mtDNA and MSY Roma South Asian lineages (M and H lineages, respectively). Markov chain Monte Carlo (MCMC) samples were based on 15,000,000 generations, sampled every 1,000, with 1,500,000 burn-in generations (10%). HKY (mtDNA) and GTR (MSY) substitution models, strick clock and bayesian skyline as evolution tree prior were selected based on the maximum marginal likelihood estimation (MLE) with the path sampling/stepping-stone sampling method implemented in BEAST2 2.5.0^75^. MCMC trace files were visualized and analyzed with Tracer 1.6^75^. mtDNA sequences were split into coding and control regions, to account for different substitution rates (1.708 × 10^−8^ and 9.883 × 10^−8^ substitutions/year/site, respectively)^73^. A fast mutation rate of 10^−9^ substitutions/year/site^74^ was used for MSY analyses. Bayesian phylogenetic trees were built with all Roma samples and node coalescence ages were estimated with BEAST2 2.5.0^75^, using the same parameters as described above. TreeAnnotator^75^ was used to summarize the sample of trees from BEAST into a consensus maximum clade credibility tree.

In both uniparental markers, Roma main haplogroups were phylogenetically refined by retrieving informative unidentified polymorphic sites with homemade scripts. The nomenclature used for the MSY haplogroups analysis was ISOGG 2019^76^.

### Ethics statement

All methods were carried out in accordance with the appropriate guidelines and regulations. DNA donors were recruited with the appropriate informed consent, and the project was reviewed and approved by the Institutional Review Board of the Comitè Ètic d’Investigació Clínica-Institut Municipal d’Assistència Sanitària (CEIC-IMAS) in Barcelona, (2016/6,723/I).

## Supplementary information

Supplementary file1

Supplementary file2

## Data Availability

mtDNA complete sequences and MSY bam files are deposited at EGA accession number: EGAS00001004207.
