# Author Correction: Sex-biased patterns shaped the genetic history of Roma

**DOI:** 10.1038/s41598-020-75277-1

**Published:** 2020-10-20

**Authors:** C. García-Fernández, N. Font-Porterias, V. Kučinskas, E. Sukarova-Stefanovska, H. Pamjav, H. Makukh, B. Dobon, J. Bertranpetit, M. G. Netea, F. Calafell, D. Comas

**Affiliations:** 1 Institute of Evolutionary Biology (UPF‑CSIC), Department of Experimental and Health Sciences, Universitat Pompeu Fabra, Barcelona, Spain; 2 Department of Human and Medical Genetics, Faculty of Medicine, Biomedical Science Institute, Vilnius University, Vilnius, Lithuania; 3 Research Center for Genetic Engineering and Biotechnology “Georgi D. Efremov”, Academy of Sciences and Arts of the Republic of North Macedonia – MASA, Skopje, Republic of North Macedonia; 4 Institute of Forensic Genetics, Hungarian Institute for Forensic Sciences, Budapest, Hungary; 5 Institute of Hereditary Pathology, Ukrainian Academy of Medical Sciences, Lviv, Ukraine; 6 Department of Internal Medicine and Radboud Center for Infectious Diseases, Radboud University Medical Center, 6525 GA Nijmegen, the Netherlands; 7 Department of Human Genetics, University of Medicine and Pharmacy Craiova, Craiova, Romania; 8 Department for Genomics and Immunoregulation, Life and Medical Sciences Institute (LIMES), University of Bonn, 53115 Bonn, Germany

Correction to: *Scientific Reports*, 10.1038/s41598-020-71066-y, published online 02 September 2020

This Article contains errors.

In the Results section under subheading ‘Roma lineages show hidden phylogenetic complexity’,

“Inside the J2a-M92 clade, we detected 5 private SNPs markers common to all 18 samples (Supplementary Table S9).”

should read:

“Inside the J2a-M67 clade, we detected 5 private SNPs markers common to all 18 samples (Supplementary Table S9).”

In addition, the Supplementary Information published with this Article contains an error in Supplementary Table S9, where,

“J2a-M92”

should read:

“J2a-M67”

Finally, in the Acknowledgements section,

“This work was supported by the Spanish Ministry of Economy and Competitiveness (grant numbers CGL2016-75389-P (MINEICO/FEDER, UE), PID2019-106485GB-I00 (MINEICO), and “Unidad María de Maeztu” (MDM-2014-0370) to DC and FC; and Agència de Gestió d’Ajuts Universitaris i de la Recerca (Generalitat de Catalunya, grant 2017SGR00702).”

should read:

“This work was supported by the Spanish Ministry of Economy and Competitiveness (grant numbers CGL2016-75389-P (MINEICO/FEDER, UE), PID2019-106485GB-I00/AEI/10.13039/501100011033 (MINEICO), and “Unidad María de Maeztu” (MDM-2014-0370) to DC and FC; and Agència de Gestió d’Ajuts Universitaris i de la Recerca (Generalitat de Catalunya, grant 2017SGR00702).”

