# Supplementary material for: Sex-biased patterns shaped the genetic history of Roma
Source: Sci Rep. 2020 Sep 2;10:14464. doi: 10.1038/s41598-020-71066-y (PMC7468237; doi:10.1038/s41598-020-71066-y)
Supplement: Supplementary file 1 — Supplementary file1 [file 41598_2020_71066_MOESM1_ESM.docx]

**Sex-biased patterns shaped the genetic history of Roma**

C.García-Fernández^1,9^, N.Font-Porterias^1,9^, V.Kučinskas^2^, E.Sukarova-Stefanovska^3^, H.Pamjav^4^, H.Makukh^5^, B.Dobon^1^ , J.Bertranpetit^1^ , MG. Netea^6,7,8^, F.Calafell^1^*, D.Comas^1^*

^1^ Institute of Evolutionary Biology (UPF-CSIC), Department of Experimental and Health Sciences, Universitat Pompeu Fabra, Barcelona, Spain

^2^ Department of Human and Medical Genetics, Biomedical Science Institute, Faculty of Medicine, Vilnius University, Vilnius, Lithuania

^3^ Research Center for Genetic Engineering and Biotechnology “Georgi D. Efremov”, Academy of Sciences and Arts of the Republic of North Macedonia – MASA Republic of North Macedonia

^4^ Institute of Forensic Genetics, Hungarian Institute for Forensic Sciences, Budapest, Hungary

^5^ Institute of Hereditary Pathology of the Ukrainian Academy of Medical Sciences, Lviv, Ukraine

^6^ Department of Internal Medicine and Radboud Center for Infectious Diseases, Radboud University Medical Center, 6525 GA Nijmegen, the Netherlands.

^7^ Department of Human Genetics, University of Medicine and Pharmacy Craiova, Romania.

^8^ Department for Genomics & Immunoregulation, Life and Medical Sciences Institute (LIMES), University of Bonn, 53115 Bonn, Germany.

* Corresponding authors

^9^ These authors contributed equally to this work.

E-mail: david.comas@upf.edu francesc.calafell@upf.edu


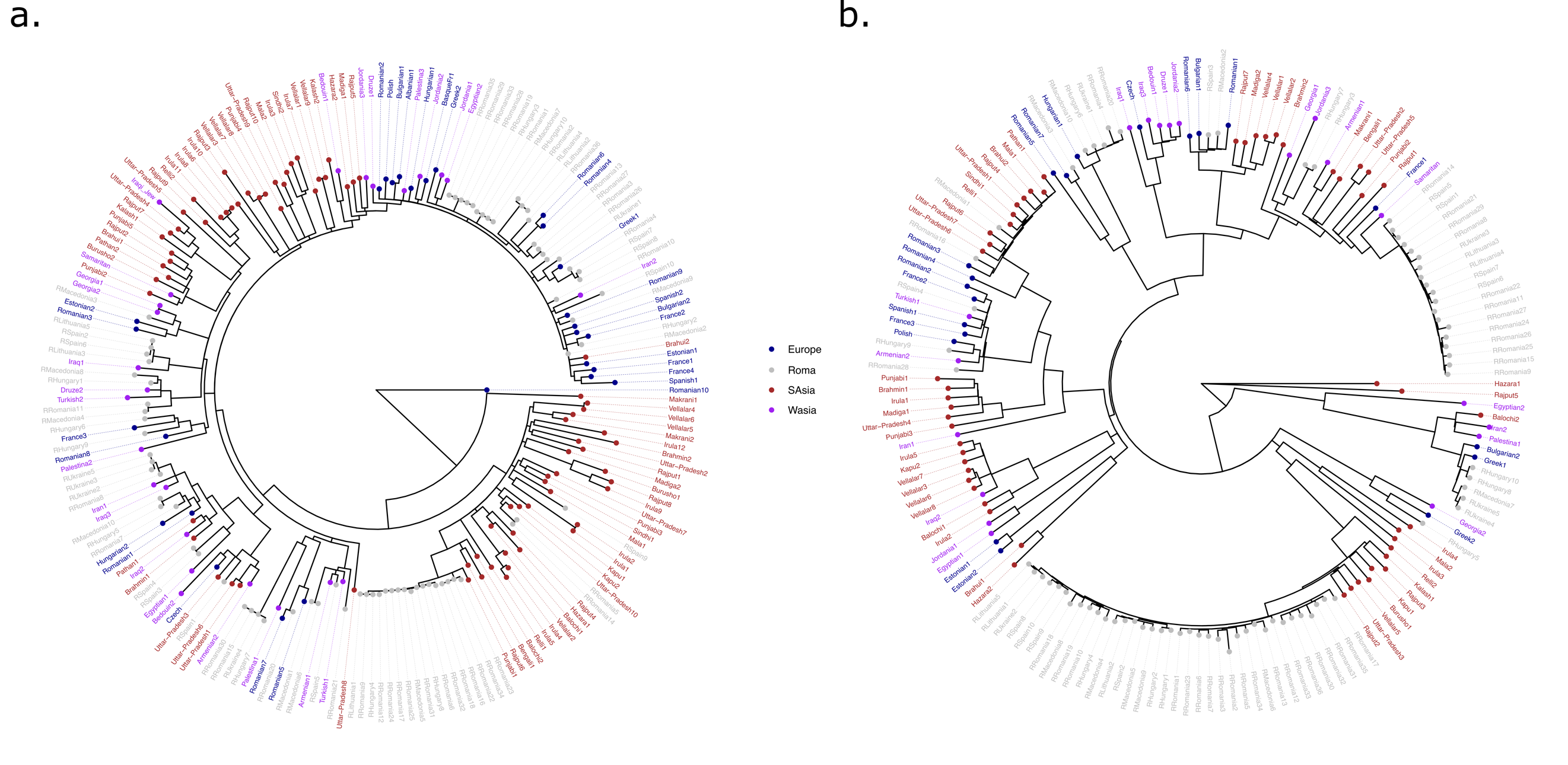


**Supplementary Figure S1.** Maximum likelihood trees for mtDNA (a) and MSY (b) sequences using Roma and non-Roma samples. Color scale refers to geographical lineage origin: blue for European, purple for West Asian, red for South Asian, and grey for Roma.


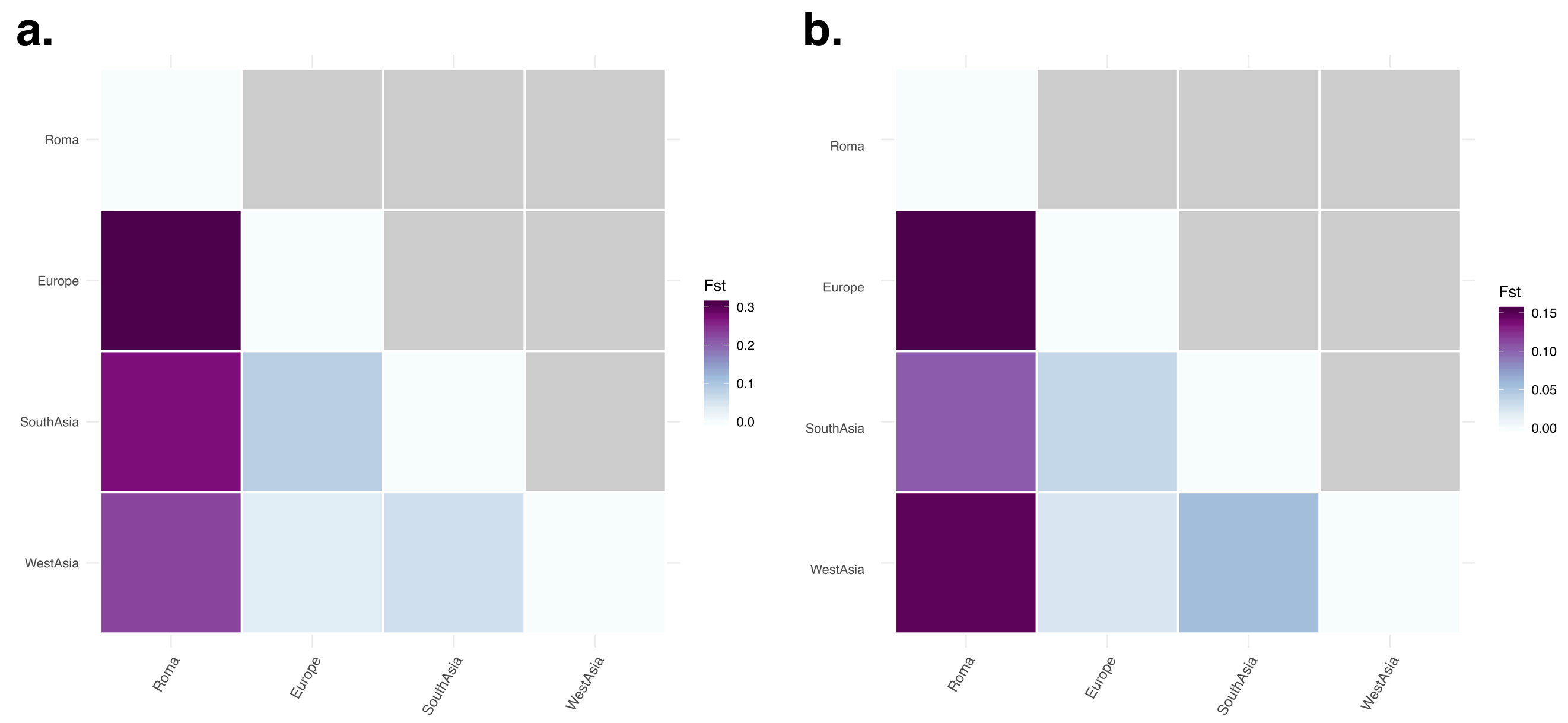


**Supplementary Figure S2**. φst distance matrices for mtDNA (a) and MSY (b) between Roma and reference panel populations. Color scales represent φst values.


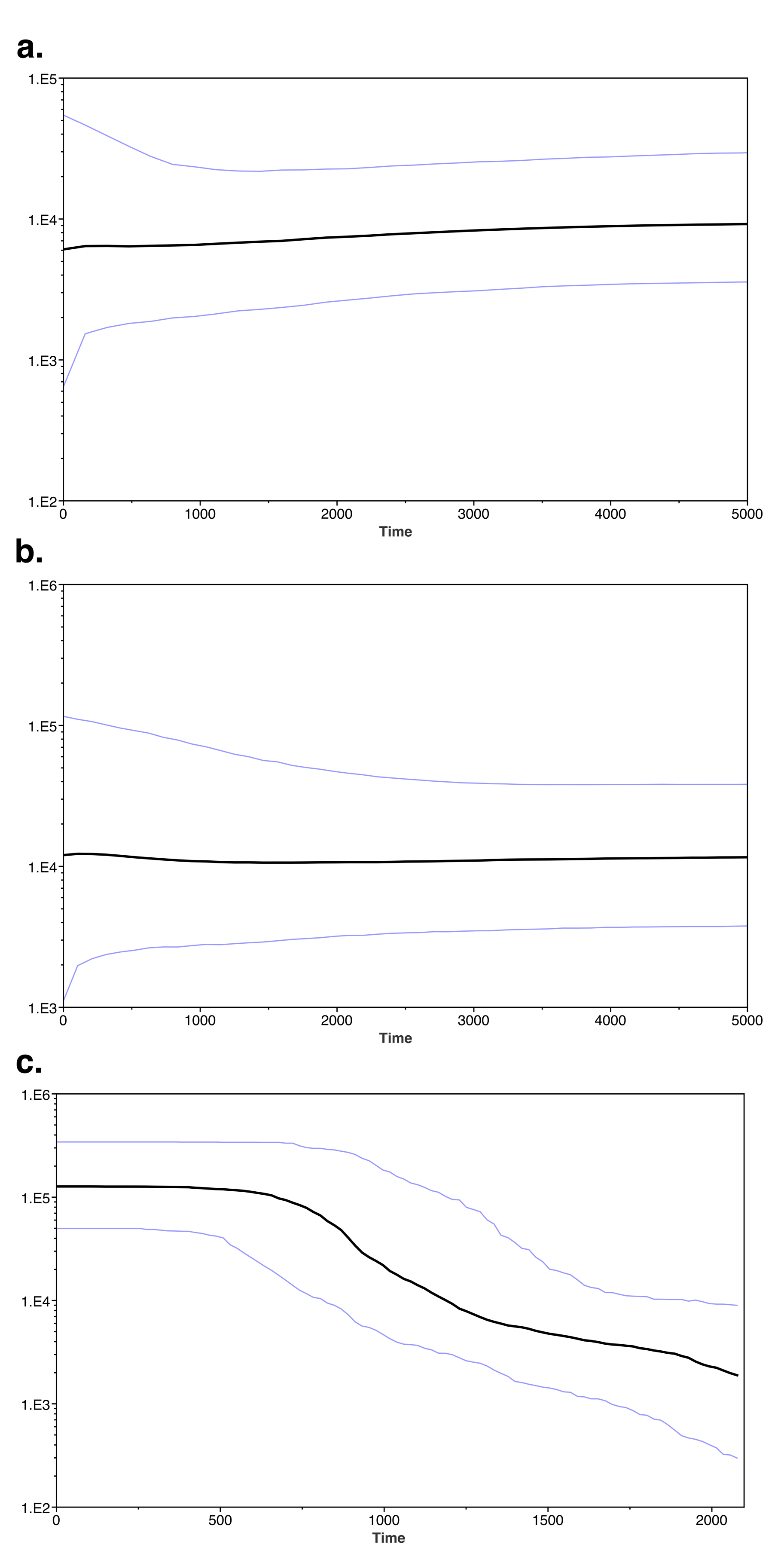


**Supplementary Figure S3.** BSP with the South Asian lineages from mtDNA coding (a) and control regions (b) and MSY sequences (c). X axis represents time in years ago.


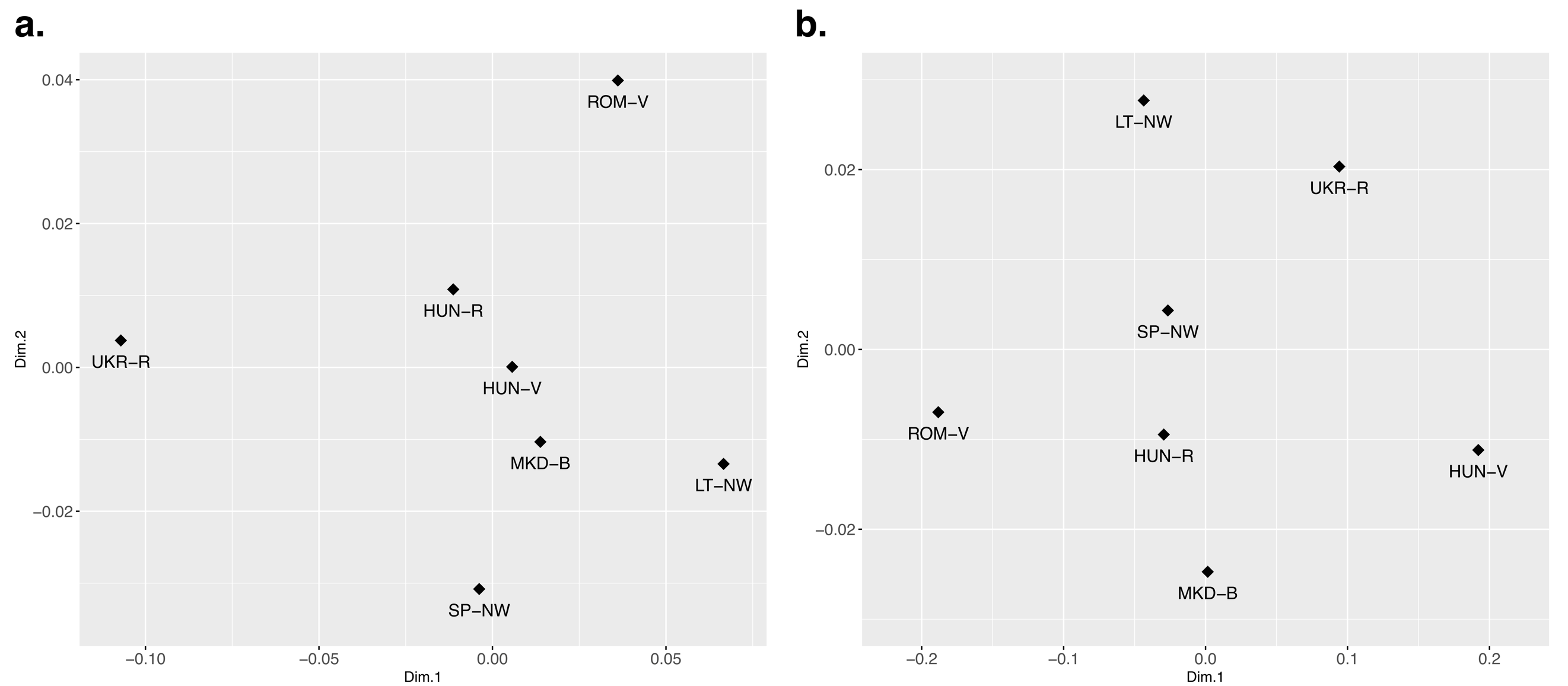


**Supplementary Figure S4.** MDS analyses from φst distances with mtDNA (a) and MSY (b) within Roma groups.


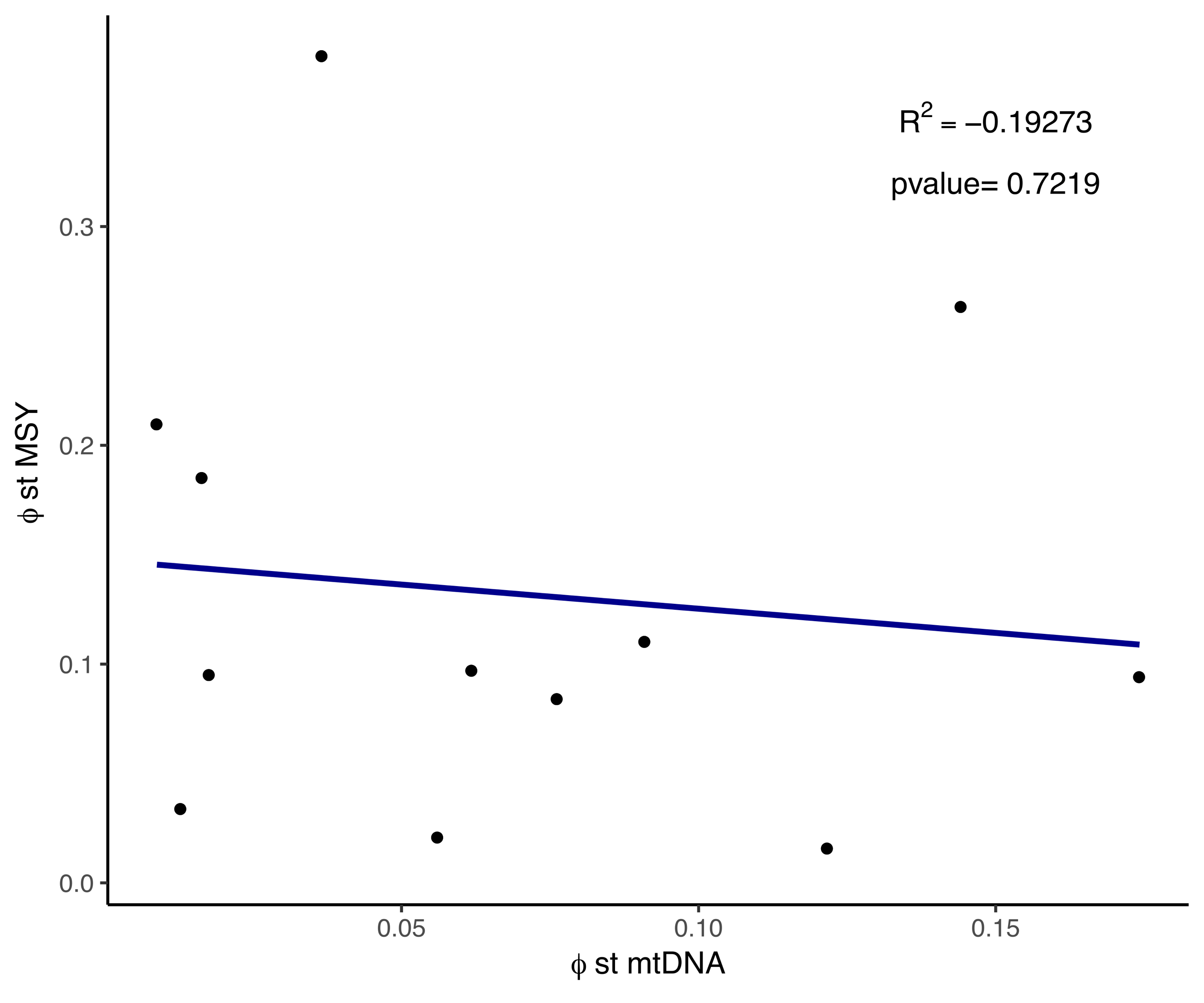


**Supplementary Figure S5.** Mantel test between mtDNA and MSY φst distances within Roma groups.


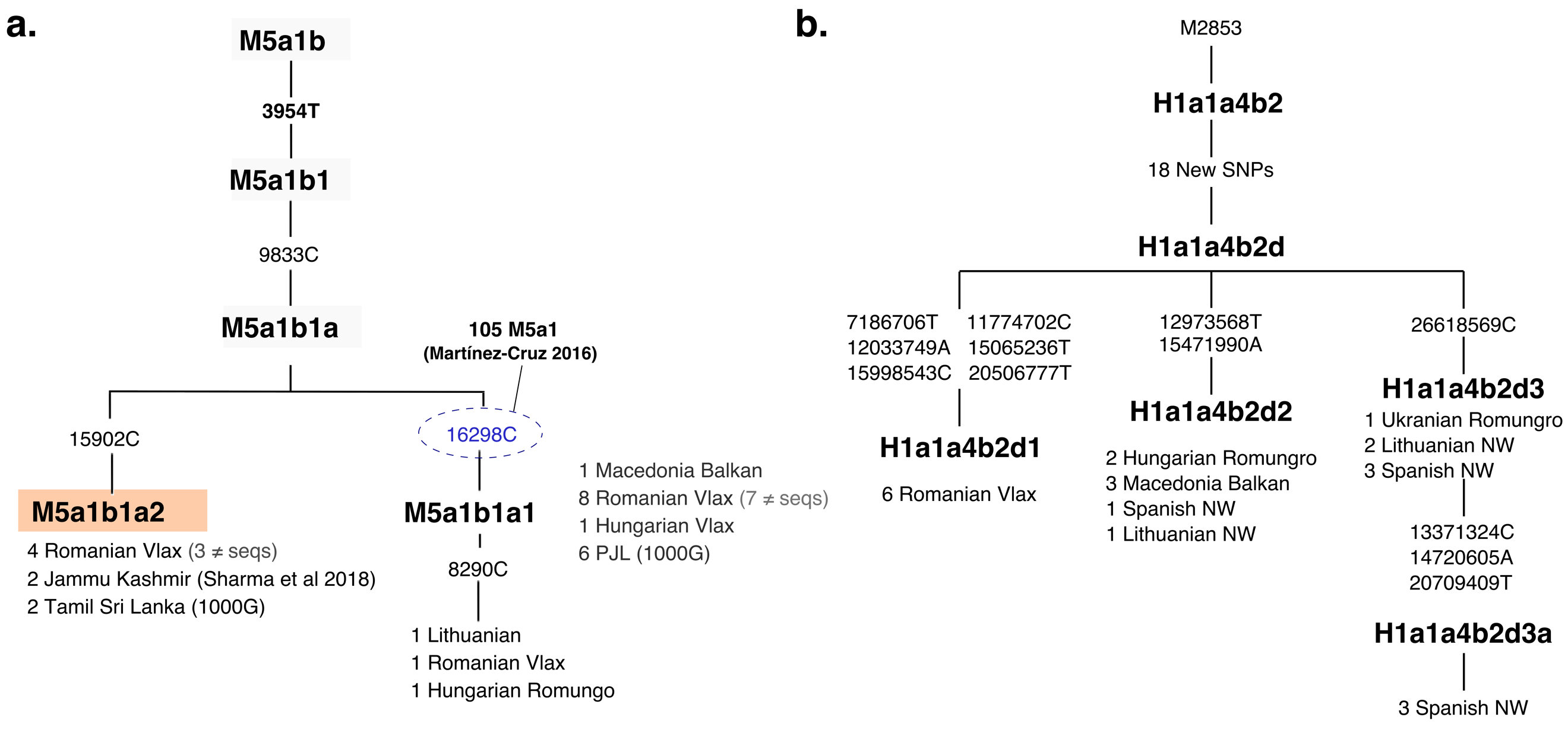


**Supplementary Figure S6.** Phylogenetic refinement of South Asian haplogroups for the mtDNA (a) and MSY (b).
